# Hypoglycemic effect of peony flowers polyphenols based on gut microbiota and metabolomics

**DOI:** 10.3389/fnut.2025.1573865

**Published:** 2025-06-02

**Authors:** Ling Chen, Xuefang Wang, Erjuan Ning, Lipan Zhang, Feifei Li, Lupeng Wang, Jie Zhu, Huanan Zhang, Tao Wang, Yanni Ma, Wei Wang, Xiao Li

**Affiliations:** ^1^Henan Napu Biotechnology Co., Ltd., Zhengzhou, China; ^2^Henan Institute of Business Science Co., Ltd., Zhengzhou, China; ^3^Institute of Chemistry Henan Academy of Sciences, Zhengzhou, China; ^4^Henan Agricultural University, Zhengzhou, China; ^5^Henan Academy of Sciences, Zhengzhou, China

**Keywords:** peony flowers polyphenols, T2DM, gut microbiota, metabolomics, SCFAs

## Abstract

**Introduction:**

Type 2 diabetes mellitus (T2DM), remains a significant global public health concern. Peony has a long history of consumption and medicinal in China, and is rich in polyphenols, flavonoids, polysaccharides, and other components. However, the hypoglycemic activity and underlying mechanism of action of peony flowers polyphenols (PP) remain nebulous. Therefore, we investigate the hypoglycemic effect and mechanism of action of PP on T2DM mice.

**Methods:**

PP was extracted and isolated from peony flowers (*Paeonia ostii* “Fengdan”), the total polyphenol content (TPC) in PP was determined by the Folin-Ciocalteu method and the contents of 17 components in PP were determined by high-performance liquid chromatography. The high-fat diet (HFD) combined with streptozotocin (STZ) was used to establish T2DM mouse model, and the hypoglycemic effect and mechanism of PP based on gut microbiota and metabolomics were investigated.

**Results:**

The TPC in PP was 81.13 ± 2.89%. The results showed that after 8 weeks of intragastric administration, PP significantly reduced the fasting blood glucose (FBG) (*P* < 0.05), serum insulin level (*P* < 0.05), and insulin resistance index (*P* < 0.05), improved impaired glucose tolerance, regulated serum liver and kidney function related indicators, significantly increased superoxide dismutase (SOD) and glutathione peroxidase (GSH-PX) levels (*P* < 0.05), significantly decreased malondialdehyde (MDA) level (*P* < 0.05) in the liver, and increased the contents of short-chain fatty acids (SCFAs) in the gut of T2DM mice. The results of *16S rRNA* sequencing showed that PP could alter the gut microbiota of T2DM mice, increase the relative abundance of Firmicutes and Bacteroidota, while decrease the relative abundance of Proteobacteria. Non-targeted metabolomics results showed that the high-dose group of PP (PPH) can reverse the metabolic disorders of metabolite markers induced by T2DM *in vivo*.

**Conclusions:**

Consequently, PP may play a hypoglycemic role by regulating intestinal flora and amino acid metabolism pathway. The research establishes a foundation for using PP as a functional food to prevent or alleviate type 2 diabetes mellitus.

## 1 Introduction

Diabetes mellitus (DM) remains a major public health challenge, posing serious risks to human health following tumors, cardiovascular, and cerebrovascular diseases. Moreover, its incidence and mortality rates are rapidly increasing globally (1). The number of patients with diabetes is projected to reach 783 million by 2045, with Type 2 diabetes mellitus (T2DM) accounting for nearly 90% of all reported cases (2, 3). T2DM is a complex endocrine, metabolic disease characterized by elevated blood glucose and abnormal blood lipid levels. Insufficient metabolic management can lead to serious long-term complications, including chronic kidney disease, retinopathy, cardiovascular disease, and neuropathy, thereby increasing the mortality rate (4, 5). Despite the available data on T2DM, its pathogenesis remains unclear. In addition to obesity, genetic factors, and pancreatic islet dysfunction, gut microbiota disorders have been linked to diabetes (6). In 2010, researchers first observed significant differences in the composition of the intestinal flora between T2DM patients and healthy individuals (7). Since then, numerous studies have demonstrated the potential contribution of gut microbiota to the onset and development of T2DM by affecting glucose and lipid metabolism, intestinal barrier function, and short-chain fatty acid levels, among other mechanisms (8–10).

Improving dietary habits, exercise therapy, and pharmacological interventions are commonly used treatments for T2DM; however, long-term drug therapy has been linked to various adverse reactions, such as gastrointestinal discomfort, hyperinsulinemia, and hypoglycemia (11–13). Traditional Chinese medicine (TCM), especially medicinal and edible herbs, have good glucose control and lowering effects, with various advantages, such as minimal toxic side effects, stable therapeutic effects, and multiple pathways and targets (14–16). Therefore, obtaining active substances with hypoglycemic properties from TCMs or using functional foods as adjunctive therapy to regulate blood glucose levels has attracted attention.

Referred to as the “King of Flowers,” peony boasts abundant resources and has a consumption and medicinal history of over a thousand years in China. According to the Compendium of Materia Medica, peony flowers have a bitter taste and neutral in nature; they harmonize, generate, and cool blood. Peony flowers are rich in polyphenols, flavonoids, polysaccharides, and other components, with antibacterial, antioxidant, anti-photoaging, anti-aging, α-glucosidase and α-amylase inhibitor activities, among other properties (17–21). Polyphenols, as a functional factor, have antioxidant, anti-inflammatory, whitening, and hypoglycemic activities (22–25), and are widely used in functional foods, pharmaceuticals, and cosmetics, such as tea polyphenols, apple polyphenols, etc. Our previous research has shown that peony flowers polyphenols have inhibitory effects on α-glucosidase activity (26), However, there are no relevant reports on the hypoglycemic effect and mechanism of action of PP.

In this study, we established a T2DM mouse model using HFD combined with STZ and investigated the effects of peony flowers polyphenols (PP) on the fasting blood glucose (FBG), serum insulin, glucose tolerance, serum biochemical indicators, liver oxidation indicators, and intestinal short-chain fatty acids (SCFAs) in T2DM mice. To explore the mechanism of PP in lowering blood glucose, the changes of intestinal flora in mouse cecum contents were analyzed using *16SrRNA* sequencing, and serum metabolomics was detected using liquid chromatography-tandem mass spectrometry (LC-MS/MS). This study provides novel insights into the potential application of PP in the prevention and treatment of diabetes.

## 2 Materials and methods

### 2.1 PP preparation

*Paeonia ostii* “Fengdan”, obtained from Luoyang Xianghe Peony Co., Ltd., were freeze-dried at low temperature (−50 to −60°C), crushed with a grinder, and filtered through a 40 mesh sieve. Next, 80% ethanol was added at a ratio of 1:20 and ultrasonic extraction was performed for 1 h. The sample was centrifuged for 10 min (4,200 rpm). The extraction was repeated four times, and the supernatant were pooled and concentrated under reduced pressure at a low temperature (50°C) until there was no alcohol taste and alcohol content detected as 0 through an alcohol meter. The pH was adjusted to approximately 2 with 6 mol/L hydrochloric acid solution. The D101 macroporous adsorption resin was treated with 95% ethanol and water before adding the extraction solution to it and washed with water to remove water-soluble substances, such as polysaccharides. The peony flower extract was eluted with 95% ethanol (10 times the volume of the peony flower extract). The eluent was collected, concentrated under reduced pressure to remove ethanol, and stored at −20°C after low-temperature freeze-drying. The sample was powdered before use, and the drug solution was prepared by suspending it in 0.5% carboxymethyl cellulose (CMC).

### 2.2 Detection of TPC and component content of polyphenols in PP

The total phenolic content (TPC) was determined by the Folin-Ciocalteu method (27), 1 mL of the sample and gallic acid standard solution (10, 20, 30, 40, 50 μg/mL) were reacted with 0.5 mL of Folin phenol reagent (1 mol/L) for 10 min. Then, 2 mL of 7.5% Na_2_CO_3_ solution and 6.5 mL of water were added, the solution was mixed thoroughly. The reaction was carried out at room temperature in the dark for 60 min, and the absorbance was measured at 750 nm wavelength. A standard curve [*y* = 0.011*x* + 0.022 (*R*^2^ = 0.998)] was prepared and the TPC in PP was calculated. Repeat three times.

The contents of polyphenol compounds in PP were detected using Agilent 1260 Infinity II high-performance liquid chromatography (UV detector, Agilent, USA) with Agilent Eclipse XDB-C18 (250 × 4.6 mm, 5 μm)chromatography column. The mobile phases A and B consisted of acetonitrile and 0.1% aqueous formic acid solutions, respectively. The gradient elution conditions were as follows: 0–15 min, mobile phase A increased from 10% to 15%; 15–28 min, mobile phase A increased from 15% to 18%; 28–40 min, mobile phase A was maintained at 18%; 40–45 min, mobile phase A increased from 18% to 25%; 45–65 min, mobile phase A increased from 25% to 42%; 65–75 min, mobile phase A increased from 42% to 60%.

The UV detection wavelength was set at 230 nm, flow rate at 1.0 mL/min, the column temperature at 35°C, and the injection volume was 10 μL. An appropriate amount of 17 standard products [gallic acid, methyl gallate, ethyl gallate, apigenin, isovitexin (Chengdu Pufide Biotechnology Co., Ltd.), luteoloside (Chengdu Must Biotechnology Co., Ltd.), apigetrin, vitexin glucoside, 1,2,3,6-*O*-tetragalloyl glucoside (Chengdu Zhibiaohua Pure Biotechnology Co., Ltd.), 1,2,3,4,6-*O*-pentagalloyl glucoside, astragalin (Lemaitian Pharmaceutical/Dest Biotech), benzoic acid, resveratrol, kaempferide, paeoniflorin (China Academy of Food and Drug Control), kaempferol-7-*O*-β-D-glucoside (Shanghai Aladdin Biochemical Technology Co., Ltd.), sinensis (Shanghai Yuanye Biotechnology Co., Ltd.)] were accurately weighed. Methanol was added to make the total volume of 10 mL. Mixed standard stock solution was prepared and divided into a series of concentration standard solutions, and a mixed standard curve was developed. The sample solutions were filtered through a 0.45 μm filter membrane, and analyzed using high-performance liquid chromatography (HPLC). Repeat three times. The polyphenol contents of PP were calculated using an external standard method.

### 2.3 Establishment of animal models

C57BL/6J male mice (7 weeks old, 18–20 g) were purchased from Henan Silibase Biotechnology Co., Ltd. [production license: SCXK (Yu) 2020-0005]. The mice were reared in an environment having a temperature of 25 ± 2°C, 50–60% humidity, and a light/dark cycle of 12/12 h with free eating and drinking. After 7 days of adaptive feeding, six mice were selected as the control (CON) group and given a regular diet (crude protein ≥18.0%, crude fat ≥4.0%, crude fiber ≤ 5.0%, crude ash ≤ 8.0%, calcium 1.0–1.8%, and phosphorus 0.6–1.2%), while 24 mice were fed with HFD (35% fat, 26% carbohydrates, and 26% protein). Four weeks later, all mice were fasted for 12 h with free access to drinking water. The HFD-fed mice were intraperitoneally injected with STZ solution (50 mg/kg of body weight) (Sigma-Aldrich, USA) dissolved in citric acid buffer (pH 4.2), and the control (CON) group mice were administered an equal volume of citric acid buffer for five consecutive days according to reported method (28) and pre-experiment. Tail-end blood collection was performed using a handheld blood glucose meter (China Sannuo Biosensor Co., Ltd., Changsha, China) to measure the FBG levels. If the FBG was higher than 11.1 mmol/L in the diabetic group, the mice were confirmed as successfully diabetic-induced. Mice with blood glucose levels lower than the standard were injected with STZ solution for three more days and reexamined. The modeling criteria were the same as those previously described (28). T2DM mice were stochastically divided into four groups (*n* = 6 per group): The model (MOD) and CON group mice were gavage-administered 0.5% CMC at 10mL/Kg/d, and metformin hydrochloride (MET) (Jiangsu Merck Pharmaceutical Co., Ltd.) was intragastric administered at a rate of 200 mg/kg/day, while the high- and low-dose PP groups (PPH and PPL groups) were administered PP by gavage at 600 and 400 mg/Kg/day, respectively. Treatments were administered for 8 weeks. During the experimental period, the weights and FBG of the mice were measured weekly at regular intervals.

### 2.4 Oral glucose tolerance test (OGTT)

The OGTT is widely used in clinical practice for the diagnosis of diabetes. After 8 weeks of treatment, the mice were fasted for 12 h with free access to water, following which FBG was measured. Based on the body weight, glucose solution was administered orally at a dose of 2 g/kg, and FBG was measured at 0, 0.5, 1, and 2 h to compute the area under the curve (AUC) during OGTT (29) according to Equation 1.


(1) $$\begin{array}{l} \text{ AUC }=\;0.5\;\times \;\left({\text{G}}_{\;0\text{h}}\;+\;{\text{G}}_{\;0.5\text{h}}\right)\;\times \;0.5\\ +\;0.5\;\times \;\left({\text{G}}_{\;0.5\text{h}}\;+\;{\text{G}}_{\;1\text{h}}\right)\;\times \;0.5\;+\;0.5\;\times \;\left({\text{G}}_{\;1\text{h}}+{\text{G}}_{\;2\text{h}}\right)\;\times \;1 \end{array}$$

### 2.5 Collection of mouse samples and detection of organ coefficients

The mice were fasted for 12 h and anesthetized with ether. Blood samples were collected from the eyeballs, and centrifuged at 1,500 rpm for 10 min, and the upper serum was collected and stored in a −80°C freezer. The liver and kidney tissues of treated mice were incised and examined for visible lesions. The fat was removed from each organ using tweezers and cleaned using pre-cooled physiological saline. Filter paper was used to absorb surface moisture before weighing to calculate the coefficients of each organ (30) according to Equation 2.


(2) $$\begin{array}{l} \text{ Organ coefficient }\left(\text{\%}\right)\;=\text{ organ wet weight}/\text{mouse body weight }\times \;100 \end{array}$$

Parts of the liver and kidney tissues were placed in a 4% paraformaldehyde fixing solution for hematoxylin and eosin (H&E) staining, while the remaining tissues were pre-frozen in liquid nitrogen and stored in a −80°C freezer.

The ceca of the mice were longitudinally cut open on ice to collect the cecum contents for detection of the gut microbiota.

### 2.6 Serum related index detection

Fasting insulin (FINS) content was detected using enzyme-linked immunosorbent assay (ELISA) detection kit (Shanghai Biyuntian Biotechnology Co., Ltd.) according to the manufacturer's instructions. The insulin resistance index (HOMA-IR) was calculated using Equation 3.


(3) $$\begin{array}{l} \text{HOMA}-\text{IR }=\left(\text{FBG }\times \text{ FINS}\right)/22.5 \end{array}$$

Chemray 800 fully automatic biochemical analyzer (Shandong Boke Biotechnology Co., Ltd.) was used to detect the blood lipid levels and liver and kidney functions related indicators in mice, including aspartate aminotransferase (AST), alanine aminotransferase (ALT), triglycerides (TG), total cholesterol (TC), low-density lipoproteins (LDL), high-density lipoproteins (HDL), albumin (ALB), urea (UREA), total protein (TP), and creatinine (CRE).

### 2.7 Detection of liver antioxidant-related indicators

Detection of superoxide dismutase (SOD), catalase (CAT), glutathione peroxidase (GSH), and malondialdehyde (MDA) was conducted using their respective detection kits (Nanjing Jiancheng Bioengineering Institute) following the manufacturer's instructions. Differences in antioxidant factors in the liver tissues of each group were compared.

### 2.8 Pathological analysis of mouse tissue

Pathological tissue sections of the mice's liver and kidney were observed following H&E staining. Liver and kidney tissue sections were dehydrated using an ethanol gradient and fixed in 4% paraformaldehyde solution. The transparent tissue was embedded in paraffin, sliced into approximately 4 μm thick sections, stained with H&E staining solution, washed with water, and sealed with neutral gum. The tissue sections were air-dried and observed for tissue changes under a microscope (DP-72; Olympus, Tokyo, Japan). The images were captured for further examination.

### 2.9 Detection of SCFAs in cecal contents of mice

Gas chromatography-mass spectrometry (GC-MS) was used to detect SCFAs in the cecal contents of mice, as previously described (31), with some modifications.

#### 2.9.1 GC-MS analysis conditions

##### 2.9.1.1 GC conditions

A DB-FFAP capillary column (30 m × 0.25 m × 0.25 mm) was used for GC. The temperature of the injection port was 250°C, the carrier gas was He gas, and the column flow rate was 0.98 mL/min.

##### 2.9.1.2 Chromatographic heating program

The starting temperature was 100°C (maintained for 5 min), increased to 120°C at 10°C/min (maintained for 5 min) and to 200°C at 10°C/min. The injection volume was 1.0 μL, and the split ratio was 10:1.

##### 2.9.1.3 MS conditions

The ion source was an electron impact ionization source (EI) with an ionization voltage of 70 eV, the ion source temperature was 230°C, generate positive ions, and the qualitative scanning range was m/z 30–500. A 0.3 s collection interval and quantitative scanning method of selective ion scanning were used, and an external standard method was used to calculate the SCFAs contents.

##### 2.9.1.4 Mixed standard preparation

Appropriate amounts of acetic acid, propionic acid, isobutyric acid, isovaleric acid, butyric acid, and valeric acids (ANPEL Laboratory Technologies (Shanghai) China) were weighed, A series of mixed standard solutions were prepared using methanol.

##### 2.9.1.5 Sample preparation

The cecal contents were accurately weighed into a 2 mL EP tube, to which 350 μL of methanol was added and homogenized using a homogenizer for 30 s until a uniform suspension was obtained. The pH was adjusted to 2–3 with concentrated sulfuric acid. A small amount of anhydrous sodium sulfate was added for dehydration and let stand for 10 min, filtered the samples through 0.45 μm filters and detection using GC-MS [GC-MS QP2010 Ultra GC-MS (Shimadzu Corporation, Japan)]. All procedures were performed on ice.

### 2.10 Metabolomics analysis of mouse serum

Untargeted metabolomic analysis of mouse serum was conducted using LC-MS/MS and entrusted to Shanghai Paisenuo Biotechnology Co., Ltd. for detection. The operation steps followed the method (32).

### 2.11 *16S rRNA* gene sequencing analysis

The *16S rRNA* gene sequencing analysis of mouse cecal contents was conducted by Shanghai Paisenuo Biotechnology Co., Ltd. The operation steps followed the method (33).

### 2.12 Statistical analysis

The experimental data were analyzed, processed, and plotted using SPSS17.0 and GraphPad Prism9.0. Data are expressed as mean ± SD. One-way analysis of variance (ANOVA) with Duncan'test were used to compare parameters between groups. *P*-value of < 0.05 indicates that the results are statistically significant.

## 3 Results

### 3.1 Determination TPC and component contents of polyphenols in PP

**Figure 1:**
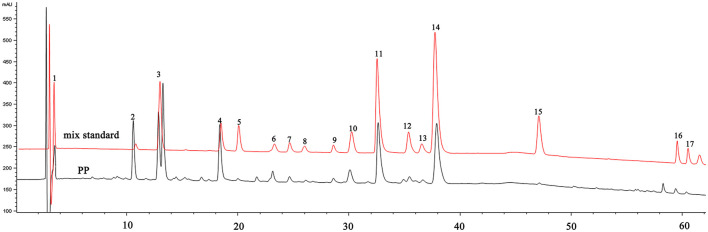
HPLC chromatogram of PP and mixed standards. *1—*Gallic acid; *2—*Methyl gallate; *3*—Sinensin; *4—*Paeoniflorin; *5—*Ethyl gallate; *6—*Vitexin glucoside; *7—*1,2,3,6-*O*-Tetragalloyl Glucose; *8*—Isovitexin; *9—*Luteoloside; *10*—Benzoic acid; *11*−1,2,3,4,6-*O*-pentagalloyl glucose; *12*—Astragalin; *13*—Kaempferol-7-*O*-glucoside; *14*—Apigetrin; *15*—Resveratrol; *16*—Apigenin; *17*—Kaempferide.

The extraction rate of PP was 3.49 ± 0.34%(w/w), and the TPC in PP was 81.13 ± 2.89%(w/w). Quantitative analysis of the content of various components in PP using HPLC and standard samples (Figure 1).The polyphenolic components of PP include phenolic acids, flavonoids, monoterpenes, tannins, and flavonoids. The percent content of each component was as follows: gallic acid, 0.18 ± 0.14%; methyl gallate, 3.86 ± 2.41%; sinensin, 6.26 ± 1.22%; paeoniflorin, 10.88 ± 0.42%; ethyl gallate, 0.17 ± 0.01%; vitexin glucoside, 1.41 ± 0.17%; 1,2,3,6-*O*-tetragalloyl glucoside, 0.61 ± 0.02%; isovitexin, 0.53 ± 0.08%; luteoloside, 0.54 ± 0.02%; benzoic acid, 1.12 ± 0.19%; 1,2,3,4,6-*O*-pentagalloyl glucoside, 10.29 ± 0.75%; astragalin, 0.99 ± 0.21%; kaempferol−7-*O*-β-D-glucoside, 0.81 ± 0.15%; apigetrin, 11.75 ± 1.03%; resveratrol, 3.54 ± 1.95%; apigenin, 0.38 ± 0.08%;kaempferide, 0.50 ± 0.15%.

### 3.2 Effects of PP on body weight, FBG, and organ index in T2DM mice

**Figure 2:**
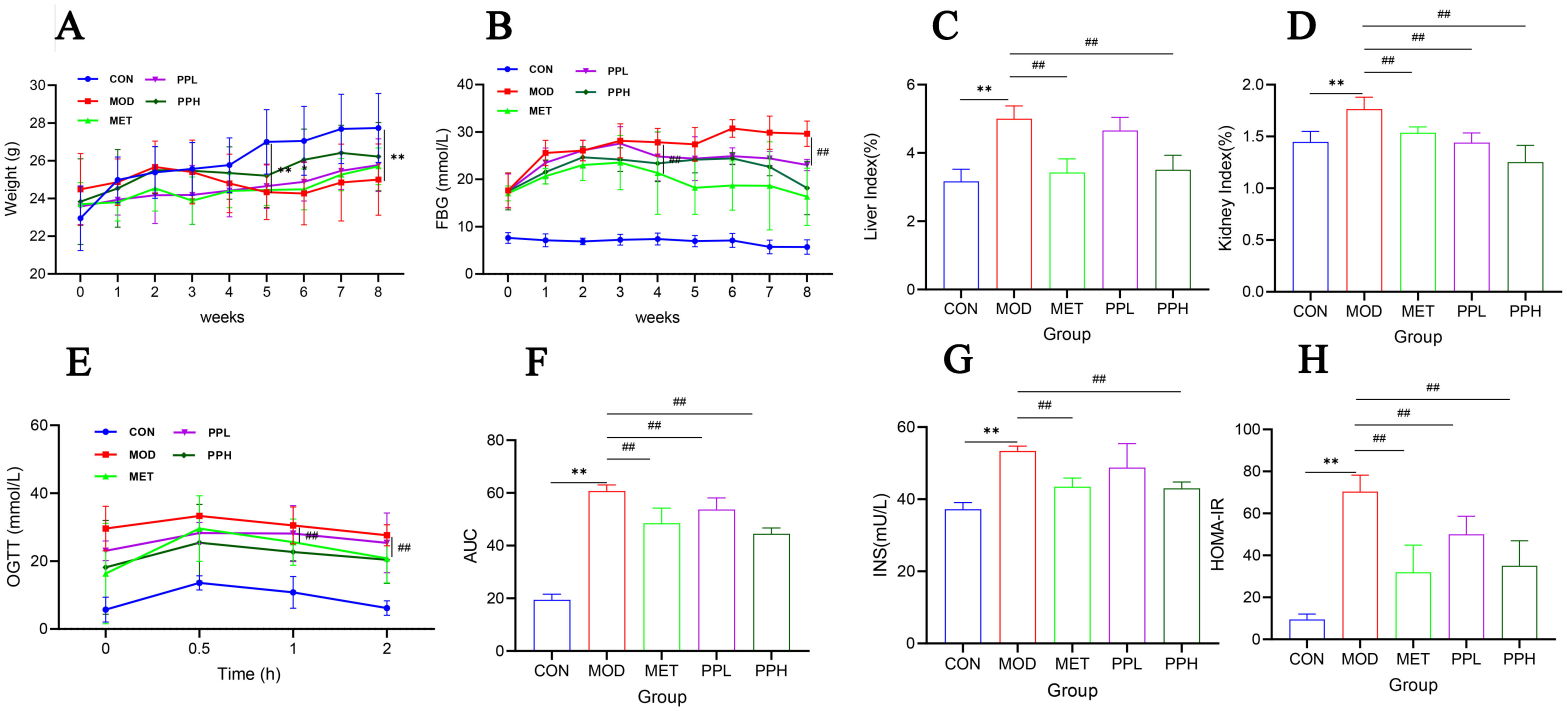
Effects of PP on body weight, blood glucose, organ index, OGTT, and serum insulin in T2DM mice. **(A)** Changes in the body weight over the 8 weeks; **(B)** Changes in the FBG levers over the 8 weeks; **(C)** Liver index; **(D)** Kidney index; **(E)** OGTT; **(F)** AUC levels of the OGTT test; **(G)** Insulin; **(H)** HOMA-IR.^**^P < 0.01 compared with the CON group; ^##^*P* < 0.01 compared with the MOD group.

The effect of PP on the body weight of T2DM mice is shown in Figure 2A. The body weight of mice in the CON group gradually increased, while that of mice in the MOD, MET, and PPH groups decreased after week 2; the body weight of mice in the MOD group was significantly lower than that in the CON group after week 5 (*P* < 0.01). The body weights of mice in the PP and MET groups gradually increased after week 5; the growth rate was relatively slow, with no significant difference between mice in the MOD and PP groups (*P* >0.05). The effects of PP on FBG in T2DM mice are shown in Figure 2B. The FBG of mice in the MOD group were significantly higher than those in the CON group (*P* < 0.01), indicating the successful modeling of T2DM. The FBG in the PPH and MET groups were lower than those in the MOD group after 8 weeks (*P* < 0.01). The results indicate that PP and MET can reduce blood glucose levels and alleviate diabetes symptoms in diabetic mice.

Compared with the CON group, the liver and kidney coefficients of the MOD group significantly increased (*P* < 0.01) (Figures 2C, D), indicating that T2DM caused liver and kidney damage. After 8 weeks of administration, the liver and kidney coefficients of all groups decreased, with the PPH group showing a significant decrease (*P* < 0.01) and better results than the MET group.

### 3.3 Effects of PP on OGTT and serum insulin in T2DM mice

OGTT is a glucose stress test used to evaluate the function of islet beta cells and the ability of the body to regulate blood glucose. As a diagnostic test for diabetes, OGTT has been widely used in clinical practice (34). The OGTT results (Figure 2E) showed that within 0.5 h of gavage administration of glucose, the FBG of each group significantly increased and then began to decrease, indicating that the body consumed the administered glucose to regulate FBG. Compared with the MOD group, the FBG levels in the PPH, PPL, and MET groups were lower. The FBG of PPH group mice was lower than that of the MOD group after 2 h (*P* < 0.01). The AUC (Figure 2F) of the MOD group was significantly higher than that of the CON group (*P* < 0.01), indicating a decrease in glucose tolerance in T2DM mice. Compared with the MOD group, the PPH, PPL, and MET groups showed a significant reduction in AUC (*P* < 0.05), indicating that PP improved the symptoms of impaired glucose tolerance in T2DM mice.

Compared to those of the CON group, the FINS level (Figure 2G) and HOMA-IR index (Figure 2H) of the MOD group significantly increased (*P* < 0.05). After 8 weeks, the PPH, PPL, and MET groups showed a significant reduction in the FINS levels and HOMA-IR (*P* < 0.05), indicating that PP enhanced insulin resistance in T2DM mice.

### 3.4 Effects of PP on serum biochemical indicators in T2DM mice

**Figure 3:**
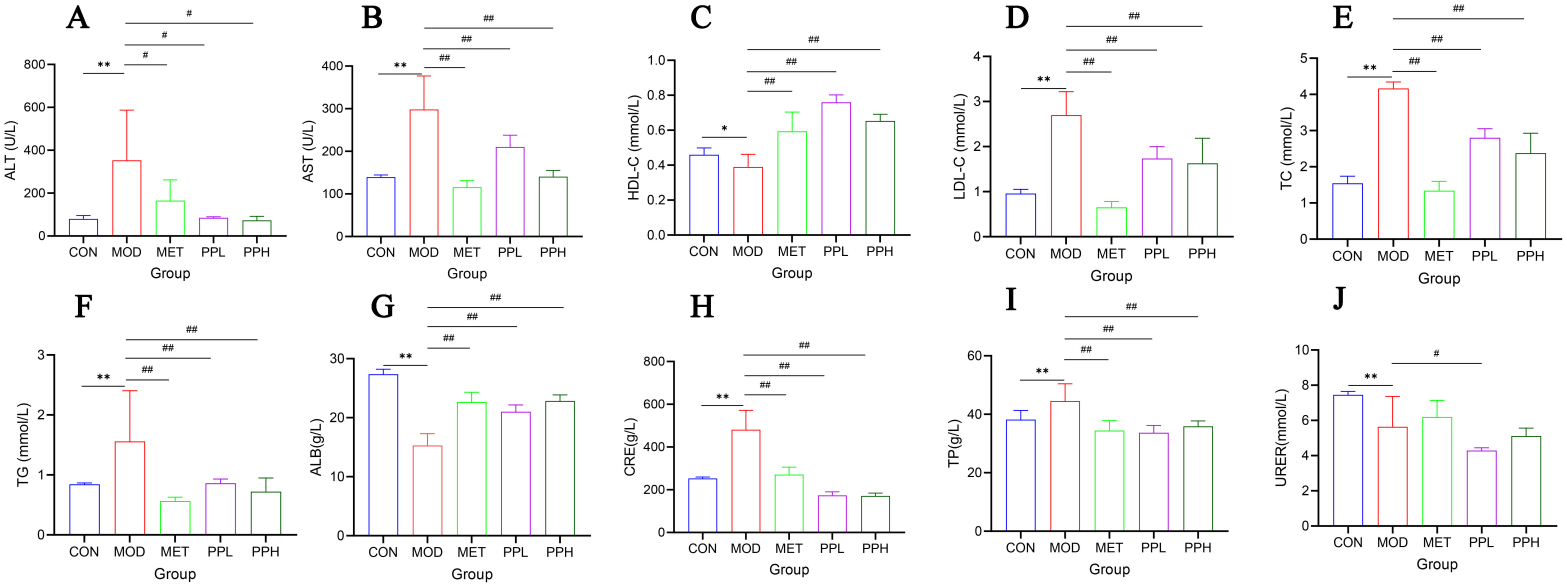
Effect of PP on serum biochemical indicators in T2DM mice. **(A)** Serum ALT levels; **(B)** Serum AST levels; **(C)** Serum HDL-C levels; **(D)** Serum LDL-C levels; **(E)** Serum TC levels; **(F)** Serum TG levels; **(G)** Serum ALB levels; **(H)** Serum CRE levels; **(I)** Serum TP levels; **(J)** Serum URER levels.**P < 0.01, *P < 0.05 compared with the CON group; ^##^*P* < 0.01, ^#^*P* < 0.05 compared with the MOD group.

As shown in Figure 3, compared to the CON group, the serum ALT, AST, TG, TC, and LDL-C levels of the MOD group mice significantly increased (*P* < 0.01), indicating that T2DM mice exhibited hyperlipidemia and liver function damage; HDL-C levels decreased, although not significantly (*P* > 0.05).

Compared with the MOD group, the PPH, PPL, and MET groups showed significantly decreased ALT, AST, TG, TC, and LDL-C (*P* < 0.01) and significantly increased HDL-C (*P* < 0.01). The results showed that PP ameliorated blood lipid levels and liver function in T2DM mice.

Compared with the CON group, the serum levels of TP and CRE in the MOD group were significantly increased (*P* < 0.01), whereas those of ALB and UREA were significantly decreased (*P* < 0.01), indicating damaged renal function in T2DM mice. Compared with the MOD group, the PPH, PPL, and MET groups showed a significant reduction in TP and CRE levels (*P* < 0.01), a significant increase in ALB (*P* < 0.01), and an increase in UREA, although not significant. This indicates that PP improves renal function in T2DM mice.

### 3.5 Effect of PP on the antioxidant capacity of the liver in T2DM mice

**Figure 4:**
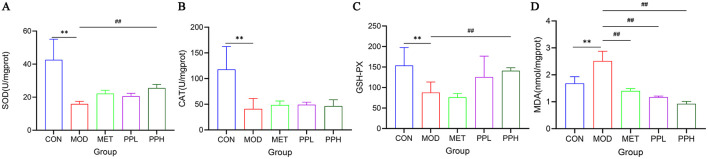
Effect of PP on MDA, GSH-PX, CAT, and MDA activities in liver tissue of T2DM mice. **(A)** Liver SOD levels; **(B)** Liver CAT levels; **(C)** Liver GSH-PX levels; **(D)** Liver MDA levels. **P < 0.01 compared with the CON group; ^##^*P* < 0.01 compared with the MOD group.

Figure 4 shows the effects of PP on the activities of SOD, GSH-Px, CAT, and MDA in the liver tissues of T2DM mice. Compared with the CON group, the levels of SOD, GSH-PX, and CAT in the livers of MOD group mice significantly decreased (*P* < 0.01), while that of MDA significantly increased (*P* < 0.01), indicating oxidative damage to the livers of T2DM mice. After 8 weeks of administration, PPH significantly increased GSH-PX and SOD levels (*P* < 0.01), increased CAT activity, and significantly decreased MDA levels (*P* < 0.01) compared with the MOD group. MDA is an index of oxidative damage and senility, whereas the other indices reflect the ability to oxidize. These results indicate that PPH enhances the antioxidant ability of T2DM mice.

### 3.6 Effects of PP on the histopathological morphology of T2DM mice tissues

**Figure 5:**
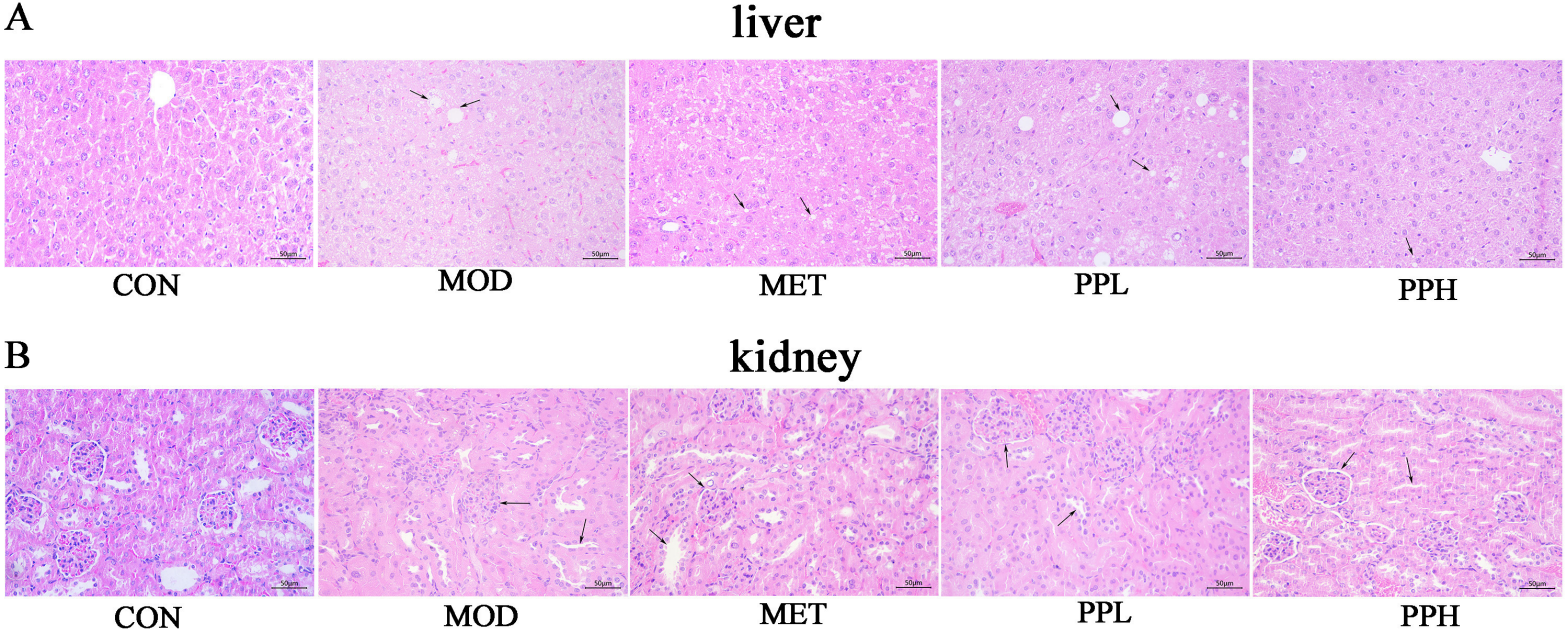
Effects of PP on the histopathological morphology of T2DM mice.

H&E staining of the liver and kidney tissues showed clear hepatic cords, regular arrangement of liver cells, and normal morphology in the CON group mice (Figure 5). The liver cells of mice in the MOD group were enlarged with unclear cell margins, disordered hepatic cord arrangement, diffuse fat-like degeneration, and necrosis. Hepatic sinusoids became smaller or disappeared. Compared with the MOD group, the liver tissue morphology and structure in the PPH, PPL, and MET groups were improved.

The renal glomeruli of mice in the MOD group were swollen and structurally unclear. Narrowing or disappearance of the renal capsule was also observed. The cortical renal tubules swelled, the lumen became smaller, and some renal tubules exhibited multifocal vacuolar degeneration and necrosis. Starch-like degeneration was observed in the collecting ducts of the renal medulla. A large amount of inflammatory cell infiltration and vascular congestion was observed in the collecting duct at the junction of the skin and marrow. The morphological structures of the collecting ducts, renal pelvis, and other tissues in the PPH, PPL, and MET groups were improved.

### 3.7 Effects of PP on the SCFAs in the cecal contents of T2DM mice

The SCFAs contents determination results are listed in Table 1. The contents of acetic acid, propionic acid, isobutyric acid, butyric acid, and isovaleric acid in the MOD group showed a decreasing trend, while that of valeric acid increased compared with the CON group. PPH and PPL increased the content of the six SCFAs, among which PPH significantly increased the contents of isobutyric acid and isovaleric acid (*P*<*0.05*) compared with the CON group.

**Table 1:** Effects of PP on SCFAs in the cecal content of T2DM mice.

**Group**	**acetic acid/μg/g**	**propionic acid/μg/g**	**Isobutyric acid/μg/g**	**butyrate/μg/g**	**Isovaleric acid/μg/g**	**Valproic acid/μg/g**
CON	373.68 ± 40.09	32.49 ± 10.16	24.33 ± 5.50	23.02 ± 11.75	10.25 ± 5.08	6.29 ± 1.94
MOD	307.27 ± 63.60^**^	14.96 ± 1.59^**^	12.63 ± 3.04^**^	11.21 ± 1.44^**^	7.31 ± 0.67	10.24 ± 0.80^**^
PPL	309.41 ± 14.44	16.55 ± 4.30	16.10 ± 2.66	11.64 ± 2.29	10.16 ± 1.50	11.54 ± 1.77
PPH	315.88 ± 7.90	18.25 ± 0.72	25.31 ± 4.53^##^	11.98 ± 1.52	11.83 ± 1.98	13.52 ± 0.59^##^
MET	314.54 ± 32.92	18.61 ± 3.17	14.98 ± 3.86	9.25 ± 4.42	16.73 ± 6.94^##^	5.20 ± 0.89^##^

^**^P < 0.01 compared with the CON group; ^##^*P* < 0.01 compared with the MOD group.

### 3.8 Effects of PP on serum metabolomics in T2DM mice

**Figure 6:**
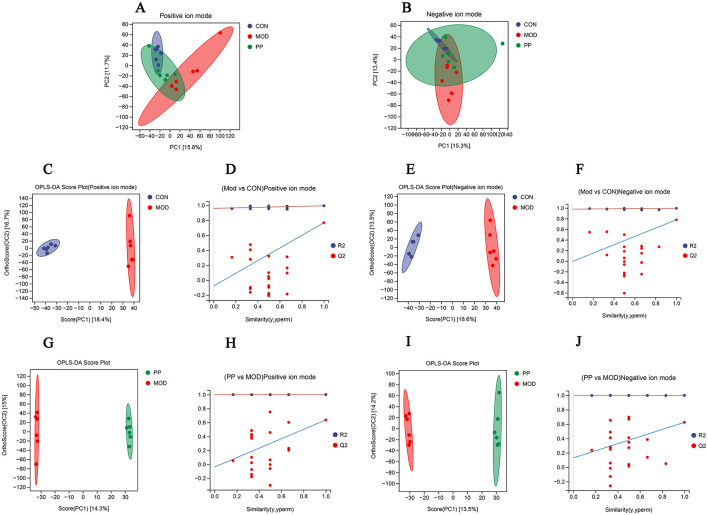
Analysis of serum metabolomics of PP in T2DM mice. **(A, B)** Scores plots of PCA between the CON, MOD, and PP groups; **(C, D)** OPLS-DA score plot and displacement test plot for the MOD and CON groups in positive ion mode; **(E, F)** OPLS-DA score plot and displacement test plot for the MOD and CON groups in negative ion mode; **(G, H)** OPLS-DA score plot and displacement test plot for the PPH and MOD groups in positive ion mode; **(I, J)** OPLS-DA score plot and displacement test plot for the PPH and MOD groups in negative ion mode.

Principal component analysis (PCA) and orthogonal partial least squares discriminant analysis (OPLS-DA) are data analysis approaches used in metabolomics to identify differences among groups. The PCA score plot (Figures 6A, B) shows a pronounced separation trend between the CON and MOD group samples in the positive and negative ion modes, and the metabolic changes in the PPH group changed toward those in the CON group.

The OPLS-DA model can achieve ideal intergroup separation and more accurately identify variables that significantly contribute to classification. To reflect the differences between groups more intuitively, an OPLS-DA analysis was conducted. The OPLS-DA analysis scores in the positive and negative ion modes are shown in Figures 6C, E, G, and I. The CON and MOD groups and the PPH and MOD groups were clearly separated in the positive and negative ion modes. The CON and MOD groups had R2Y of 0.995 and Q2 of 0.768 in the positive ion mode and R2Y of 0.995 and Q2 of 0.779 in the negative ion mode, whereas the MOD and PPH groups had R2Y of 1.000 and Q2 of 0.636 in the positive ion mode and R2Y of 0.999 and Q2 of 0.624 in the negative ion mode. This indicated good prediction ability and fitting of the model and showed differences in serum metabolites between MOD and CON mice. PPH can cause obvious metabolic changes in T2DM mice. The availability of the OPLS-DA model was verified using 200 permutation tests (Figures 6D, F, H, and J). The Q2 point on the far-right side of the OPLS-DA permutation test chart was higher than the other points, indicating no overfitting in this model.

**Figure 7:**
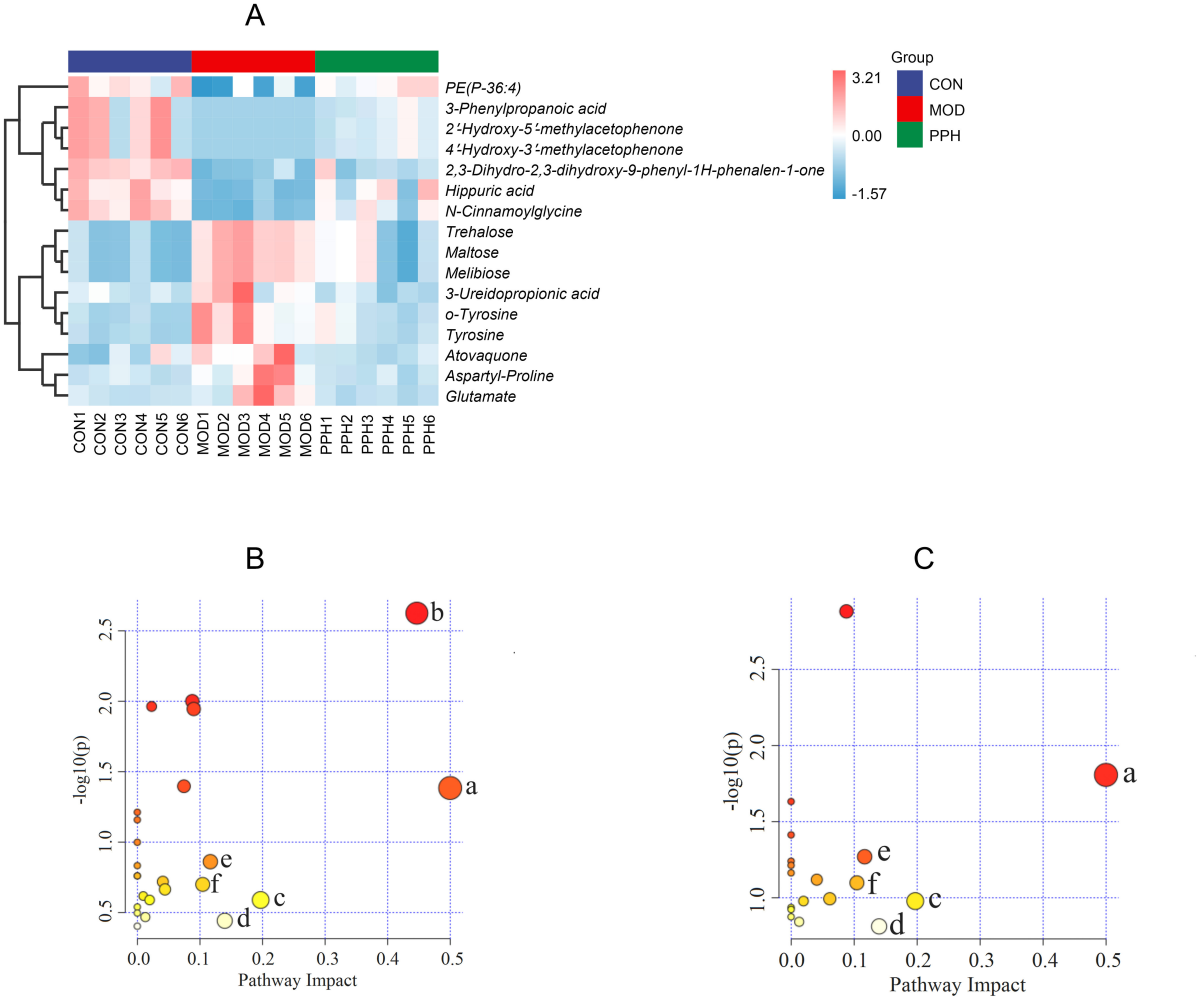
**(A)** Differential metabolite heatmap; **(B)** KEGG plot of MOD vs. CON; **(C)** KEGG plot of PPH vs. MOD. (a) Phenylalanine, tyrosine and tryptophan biosynthesis; (b) Galactose metabolism; (c) Alanine, aspartate and glutamate metabolism; (d) Tyrosine metabolism; (e) Arginine biosynthesis; (f) β-Alanine metabolism.

The serum differential metabolites in the CON and MOD groups, and PPH and MOD groups were compared using the OPLS-DA model. The selection criteria were variable importance in the projection (VIP) > 1.0, fold change (FC) > 2.0, and FC < 0.5 (35, 36). There were 80 differential metabolites (30 upregulated and 50 downregulated) between the MOD and CON groups and 30 between the PPH and MOD groups (10 upregulated and 20 downregulated). A comparison of the changes in the relative contents between the groups showed that the relative contents of 16 differential metabolites were corrected following treatment with PP. The relative content heatmap of the 16 metabolites is shown in Figure 7A, and the information on these differential metabolites is listed in Table 2. These findings suggest that PP can reverse metabolic disorders of metabolites induced in T2DM mice *in vivo*.

**Table 2:** The differential metabolites in serum after PPH treatment.

**No**.	**HMDB**	**Metabolites**	**Mode**	**VIP**		**FC**		**Trend**	
				**CON vs MOD**	**MOD vs PP**	**CON vs MOD**	**MOD vs PP**	**CON vs MOD**	**MOD vs PP**
1	HMDB0000026	3-Ureidopropionic acid	POS	1.40	1.87	2.01	0.41	↑^*^	↓^**^
2	HMDB0000048	Melibiose	POS	2.17	1.90	3.18	0.47	↑^**^	↓^**^
3	HMDB0000148	Glutamate	NEG	1.61	2.01	2.59	0.34	↑^*^	↓^**^
4	HMDB0000158	Tyrosine	POS	1.89	1.71	3.04	0.47	↑^**^	↓^*^
5	HMDB0000163	Maltose	POS	2.17	1.90	3.18	0.47	↑^**^	↓^**^
6	HMDB0000714	Hippuric acid	NEG	2.19	2.16	0.11	6.32	↓^**^	↑^**^
7	HMDB0000764	3-Phenylpropanoic acid	NEG	2.29	2.29	0.00	4,640.80	↓^**^	↑
8	HMDB0000975	Trehalose	POS	2.17	1.90	3.17	0.47	↑^**^	↓^**^
9	HMDB0006050	o-Tyrosine	POS	1.90	1.74	3.11	0.45	↑^**^	↓^*^
10	HMDB0011352	PE(P-36:4)	POS	1.79	1.94	0.40	2.15	↓^**^	↑^**^
11	HMDB0011621	N-Cinnamoylglycine	NEG	2.12	2.19	0.10	5.17	↓^**^	↑^**^
12	HMDB0015249	Atovaquone	NEG	1.44	2.07	2.72	0.31	↑^*^	↓^*^
13	HMDB0028761	Aspartyl-Proline	POS	1.57	1.77	2.27	0.42	↑^*^	↓^*^
14	HMDB0032592	2'-Hydroxy-5'-methylacetophenone	NEG	2.29	2.29	0.00	4,640.59	↓^**^	↑
15	HMDB0034688	2,3-Dihydro-2,3-dihydroxy-9-phenyl-1H-phenalen-1-one	POS	2.15	1.55	0.17	2.27	↓^**^	↑
16	HMDB0059824	4'-Hydroxy-3'-methylacetophenone	NEG	2.29	2.29	0.00	4,602.81	↓^**^	↑

“↑” indicates up-regulation, “↓” indicates down-regulation. ^**^P < 0.01, ^*^P < 0.05.

To gain deeper insights into the changes in metabolites and metabolic pathways in T2DM mice following treatment with PP, the differential serum metabolites between the MOD and PPH groups were imported into MetaboAnalyst 6.0 for metabolic pathway analysis (Figures 7B, C). Metabolic pathways with an impact factor >0.10 were selected as significant. Compared with the CON group, the significantly altered metabolic pathways in the MOD group mice included phenylalanine, tyrosine, and tryptophan biosynthesis, galactose metabolism, alanine, aspartate and glutamate metabolism, and tyrosine metabolism, arginine biosynthesis, and β-alanine metabolism. Compared with the MOD group, the metabolic pathways significantly regulated by PP included phenylalanine, tyrosine, and tryptophan biosynthesis, alanine, aspartate, and glutamate metabolism, tyrosine metabolism, arginine biosynthesis, and β-alanine metabolism.

### 3.9 Effects of PP on gut microbiota in T2DM mice

To determine whether the hypoglycemic effects of PP are associated with the gut microbiota, the *16S rRNA* gene V3-V4 region sequencing of the intestinal flora in the cecal contents of T2DM mice was performed after 8 weeks of treatment.

**Figure 8:**
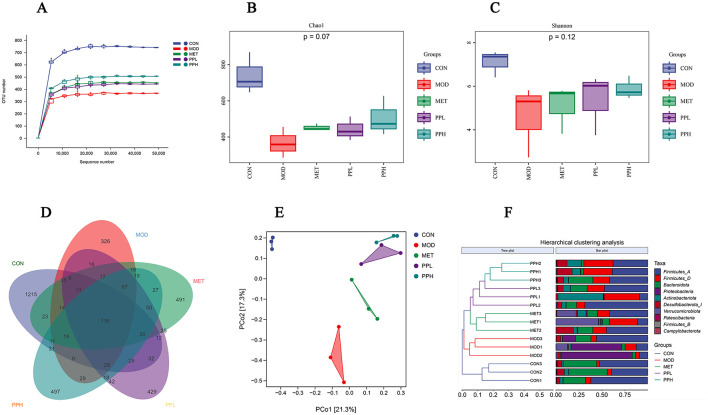
Effects of PP on gut microbiota in T2DM mice. **(A)** OTU sparse curve; **(B)** Chao index of OTU level; **(C)** Shannon index of OTU level; **(D)** Venn diagram based on OTU; **(E)** PCoA analysis of mouse gut microbiota; **(F)** Hierarchical clustering analysis diagram at the door level.

As shown in Figure 8A, the sparse curve indicated that each detected sample had an average sequencing depth of 50,000. After the sequencing depth reached 10,000, the upward trend of the curve gradually slowed, eventually approaching a straight line, indicating that the current data and sample size truly reflected the species diversity.

The α-diversity analysis results are shown in Figures 8B, C. The chao1 index reflects species abundance, whereas the Shannon index represents species diversity. As shown in Figure 8, the chao1 and Shannon indices of the MOD group were significantly lower than those of the CON group (*P* < 0.05), implying a significant decrease in the diversity and richness of the gut microbiota in the T2DM mice.

Compared with the MOD group, the chao1 and Shannon indices of the PPH, PPL, and MET groups increased (*P* > 0.05).

Venn diagrams can be used to determine the number of shared and unique operational taxonomic units (OTU) in a sample, visually displaying the similarity and overlap of OTUs in different environmental samples (37). The Venn diagram analysis conducted on the microbial composition of the MOD and PP groups (Figure 8D) showed 118 common OTUs among the 5 groups, 1 215 unique OTUs in the CON group, 326 OTUs in the MOD group, 491 OTUs in the MET group, and 497 and 429 OTUs in the PPH and PPL groups, respectively. This indicates that PP administration for 8 weeks increases the unique OTUs number and diversity of the intestinal flora in T2DM mice.

To investigate the differences in intestinal flora between different groups and determine the influence of different treatments on the structure of intestinal flora, we performed β-diversity diversity analysis. The PCoA results (Figure 8E) showed that the samples from the MOD and CON groups completely separated, indicating a significant difference in the form of intestinal flora between these groups. The PPL, PPH, MET, and MOD groups were distinguishable. The results of the hierarchical clustering analysis at the phylum level (Figure 8F) showed a significant separation in the gut microbiota of the MOD and CON groups. The gut microbiota of the PPH and PPL groups were clustered together and significantly separated from that of the MOD group, indicating that PP can regulate the intestinal flora of T2DM mice.

**Figure 9:**
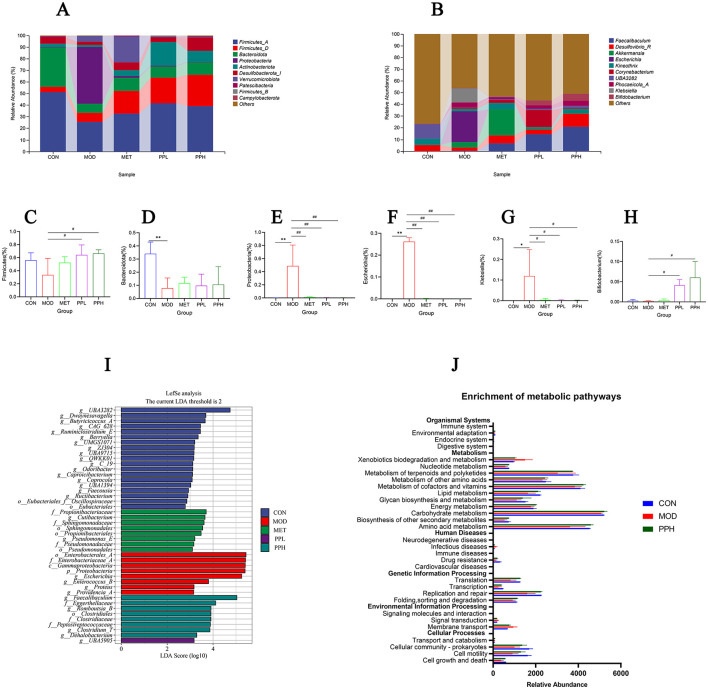
Analysis of gut microbiota structure and species differences. **(A)** Percent of community abundance on phylum level; **(B)** Percent of community abundance on genus level; **(C)** Bacteroidota; **(D)** Firmicutes(the sum of Firmicutes_A, Firmicutes_B, and Firmicutes_D)*;* **(E)** Proteobacteria; **(F)** *Escherichia*;**(G)** *Klebsiella*; **(H)** *Bifidobacterium*; **(I)** LDA scoring plot based on LEfSe analysis; **(J)** The differential metabolic pathways of PPH on T2DM were predicted using PICRUSt analysis based on the *16SrRNA* sequencing data, CON, MOD, and PPH groups. **P < 0.01, *P < 0.05 compared with the CON group; ^##^*P* < 0.01, ^#^*P* < 0.05 compared with the MOD group.

Figure 9A shows the relative abundance of the top 10 gut microbiota with advantages at the phylum level for each group. Compared with the CON group, the relative abundances of Bacteroidota and Firmicutes significantly decreased (*P* < 0.05), while that of Proteobacteria significantly increased (*P* < 0.05) in the MOD group. The PPL and PPH groups showed an increased relative abundance of Firmicutes (Figure 9C) and Bacteroidota (Figure 9D), whereas PPH, PPL, and MET significantly reduced the relative abundance of Proteobacteria (Figure 9E, *P* < 0.05).

At the genus level (Figure 9B), the relative abundance of *Escherichia* and *Klebsiella* in the MOD group significantly increased (*P* < 0.05), while that of *Bifidobacterium* decreased compared with those in the CON group. PPH significantly reduced the relative abundance of *Escherichia* (Figure 9F) and *Klebsiella* (Figure 9G) (*P* < 0.05) and significantly increased that of *Bifidobacterium* (Figure 9H) (*P* < 0.05).

The discrepancy in the functional composition of the gut microbiota among the groups was further explored using linear discriminant analysis effect size (LEfSe) analysis [linear discriminant analysis (LDA) score >4]. Figure 9I shows significant differences in the types of gut microbiota among groups. At the phylum level, the CON group showed differences in gut microbiota; the biomarker species belonged to the phylum Firmicutes, while those in the MOD group belonged to the phylum Proteobacteria. The PPH group showed differences in the gut microbiota, with biomarker species belonging to Firmicutes and Actinobacteria.

Phylogenetic investigation of communities by reconstruction of unobserved states (PICRUSt) was used to analyze and predict the effects of PP on intestinal flora function in T2DM mice. Prediction results based on the KEGG database are shown in Figure 9J. PPH significantly improved the intestinal flora of T2DM mice, making them more inclined toward the CON group. The metabolic pathways with more obvious changes included amino acid metabolism, terpenoid and polyketide metabolism, and replication and repair pathways.

**Figure 10:**
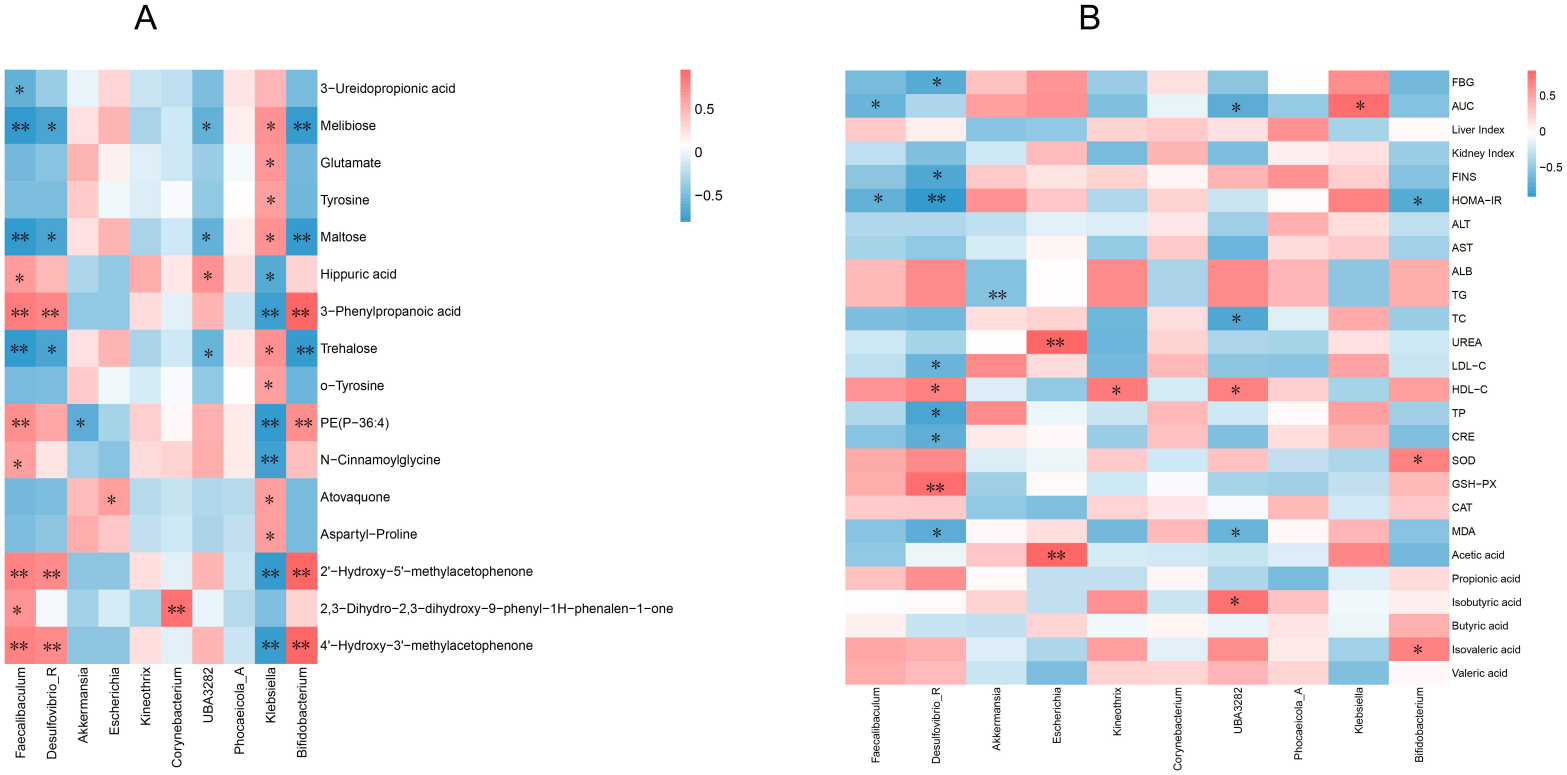
Correlation analysis. **(A)** Correlation analysis between level gut microbiota and differential metabolites; **(B)** Correlation analysis of intestinal microflora at genus level and diabetes index components. **P < 0.01, *P < 0.05 compared with the CON group.

Pearson's correlation analysis was conducted on the 10 most abundant gut microbiota at the genus level; 16 differentially expressed metabolites were highly correlated with PP treatment (Figure 10A) and diabetes index components (Figure 10B). The results showed that *Faecalibaculum, Desulfovibrio R, UBA3282, Klebsiella*, and *Bifidobacterium* were significantly positively or negatively correlated with most metabolites, indicating that the regulatory effects of PP on serum metabolites were associated with changes in the gut microbiota. *Faecalibaculum, Desulfovibrio_R, UBA3282*, and *Bifidobacterium* were negatively correlated with most index components of diabetes, such as blood glucose level, insulin resistance index, and TG, and positively correlated with SOD, GSH-PX, CAT, and other components. Among these, *Desulfovibrio_R* and *UBA3282* negatively correlated with most of the index components of MOD (*P* < 0.05), indicating that these gut microbial species could contribute to alleviating MOD symptoms.

## 4 Discussion

T2DM is a metabolic disease characterized by insulin resistance and hyperglycemia, and it is often accompanied by a disorder of lipid metabolism (38, 39).In this study, we found that PP could reduce FBG, liver and kidney coefficients, serum insulin, and insulin resistance index and improve the impaired glucose tolerance of T2DMmice after 8 weeks of intragastric administration PP (600 and 800 mg/kg), In addition, PP significantly reduced the levels of TG, TC, and LDL-C, and increased the level of HDL-C in the serum of T2DM mice, indicating that PP have the effect of reducing blood glucose and blood lipids, improving insulin resistance in T2DM mice.

High fat and glucose levels decrease the body's total antioxidant capacity, resulting in lower SOD and GSH-Px contents, aggravating oxidative stress, islet cell damage, and diabetes (40). Therefore, oxidative stress is common in patients with T2DM and insulin resistance and animal models (41, 42). In this study, we found that PPH significantly increased the levels of SOD and GSH-PX (*P* < 0.01), increased the level of CAT, and significantly reduced the level of MDA (*P* < 0.01) in the livers of T2DM mice. Additionally, the liver, the primary organ involved in glucose metabolism, is susceptible to diabetes, resulting in liver damage. Rascon et al. (43) confirmed that targeted regulation of the oxidative stress response can effectively reduce liver damage and offer liver protection. Patients with diabetes have a high blood glucose level for an extended period, which can increase the burden on the kidneys, thus leading to renal damage and decline in function. This study found that PP reduced the levels of ALT, AST, TP, and CRE in the serum of diabetic mice (*P* < 0.01), significantly increased the level of ALB (*P* < 0.01), and improved the morphological structure of the liver and kidney tissue in T2DM mice. These results suggest that PP may alleviate liver and kidney damage in T2DM mice by enhancing their antioxidant capacity and reducing oxidative stress.

The gut microbiota is an important component of the host's gut. Intestinal flora disorders are associated with diabetes, and intestinal microecology disorders induce insulin resistance and metabolic endotoxemia related to T2DM (44). In this study, the α-diversity and Venn diagram showed that the richness, diversity, and species number of intestinal flora in the MOD mice were significantly reduced, which were significantly restored following administration of PP.

Bacteroidetes can degrade or ferment dietary fiber, produce short-chain fatty acids, improve host immunity, and maintain the intestinal microecological balance (45). The abundance of Bacteroidetes in T2DM patients significantly decreases (46). Larsen et al. (7) investigated the differences in intestinal flora composition between T2DM and non-T2DM individuals and found that compared with non-diabetic individuals, the abundance of Firmicutes in T2DM individuals was significantly reduced. There were also studies indicating that HFD can reduce the abundance of Firmicutes related bacteria (47). This study found that the relative abundance of Bacteroidetes and Firmicutes in T2DM mice decreased, consistent with the above research results, and increased after intervention with PP.

*Bifidobacterium* are the most commonly used beneficial bacteria in diabetes research. The proportion of *Bifidobacterium* is significantly reduced in patients with diabetes mellitus(48). *Bifidobacterium* can exert hypoglycemic effects by reducing MDA and reactive oxygen species (ROS) levels, increasing antioxidant enzyme levels, and regulating insulin secretion in T2DM rats (49) and is also involved in acetic acid metabolism (50). The authors found that the gut microbiota composition of patients with diabetes differed from that of healthy individuals, with *Escherichia* being relatively abundant. *Escherichia coli* in *Escherichia* significantly increases the host body weight and aggravates obesity, impairing glucose tolerance (51). *Klebsiella pneumoniae*in *Klebsiella* is easy to colonize in diabetes patients and cause infection. The vascular intima of patients with diabetes is damaged due to the high-glucose internal environment, resulting in microangiopathy (52). *Klebsiella pneumoniae* was the dominant bacterial isolate from urine of T2DM patients with urinary tract infections (53). Therefore, *Escherichia* and *Klebsiella* are harmful microbiome to patients with diabetes. The correlation analysis showed that most diabetes indicators, such as blood glucose, insulin resistance index, and triglycerides, were negatively correlated with *Bifidobacterium* and positively with *Escherichia* and *Klebsiella* (Figure 10B). The analysis indicated that the hypoglycemic mechanism of PP is closely related to gut microbiota.

SCFAs are the primary metabolites produced during the fermentation of carbohydrates, proteins, and amino acids by the gut microbiota and regulate FBG levels and lipid metabolism, increase nutrition, and ameliorate insulin sensitivity. In patients with T2DM, bacteria that produce SCFAs is significantly reduced (54). This study found that PPH increased the levels of acetic acid, propionic acid, butyric acid, and valeric acid in T2DM mice and significantly increased the levels of isobutyric acid and isovaleric acid (*P* < 0.05). Correlation analysis showed that *UBA3282*, and *Bifidobacterium* were positively correlated with isobutyric and isovaleric acids, while *UBA3282* were negatively correlated with most indicators of T2DM (Figure 10B). This indicated that PP might alleviate symptoms in T2DM mice by regulating SCFAs production by gut microbiota.

Metabolomics can quantitatively and qualitatively analyze all endogenous small molecule metabolites in organisms, identifying the correlation between dynamic changes in metabolites and the physiology and pathology of diseases (55), Therefore, it has been widely used in the research of cardiovascular diseases, diabetes and other fields (56, 57). The pathway enrichment results of differential metabolites indicated that PPH significantly regulates Amino acid metabolism pathways, Tyrosine metabolism. Tyrosine is an aromatic amino acid, the study found that high levels of branched chain amino acids and aromatic amino acids are closely related to the induction of precursor diabetes and type 2 diabetes, which can be used as risk factors for predicting type 2 diabetes (58). Sun et al. (59) conducted a meta-analysis of 46 relevant studies and found that an increase in tyrosine and phenylalanine metabolic pathways are positively correlated with the risk of developing T2DM. Sriboonvorakul et al. (60) found that glutamic acid was significantly associated with T2DM. The analysis results of this study showed that the levels of tyrosine and glutamate in the serum of the MOD group mice were significantly increased compared with the CON group, which is consistent with the above research, After PPH intervention, the levels of tyrosine and glutamate in the serum of T2DM mice were significantly reduced, indicating that PP may lower blood glucose levels in T2DM mice by regulating amino acid metabolism pathways.

Intestinal flora are involved in the metabolism and utilization of amino acids, which generate metabolites that potentially affect human health. The predictive analysis of gut microbiota showed that the PPH and MOD groups significantly altered metabolic pathways such as amino acid metabolism, etc. Correlation analysis showed that *Bifidobacterium*, which is beneficial for T2DM, negatively correlated with tyrosine and glutamic acid levels. These results suggest that PP may exert hypoglycemic effects by regulating the composition of gut microbiota and amino acid metabolism pathways.

The chemical components with high content in PP include paeoniflorin, 1,2,3,4,6-*O*-pentagalloyl glucoside, apigetrin, etc. Paeoniflorin not only has hypoglycemic effect, but also can improve complications of diabetes, such as type 2 diabetes nephropathy and diabetic cardiomyopathies (61–63). 1,2,3,4,6-*O*-pentagalloyl glucoside has the effects of lowering blood glucose, inhibiting adipogenesis, and alleviating insulin resistance (64, 65). The above shows that paeoniflorin and 1,2,3,4,6-*O*-pentagalloylglucoside are the main chemical components of PP to reduce blood glucose, and can be used as potential adjuvant drugs for diabetes and its complications. These findings provide a reference for future research on PP treatment or adjuvant treatment mechanism of diabetes.

## 5 Conclusions

PP can reduce blood glucose and lipid levels in T2DM mice and reduce symptoms of T2DM in mice. Its mechanism may be related to altering the distribution of gut microbiota in T2DM mice, leading them to develop in a beneficial direction, reversing the metabolic disorders of metabolic markers induced by T2DM, and regulating amino acid metabolism pathways. Then, as the hypoglycemic mechanism of PP involves multiple components and targets, further verification and research through fecal transplantation and other techniques is needed

## Data Availability

The raw data supporting the conclusions of this article will be made available by the authors, without undue reservation.
